# Oncolytic Herpes Simplex Virus Encoding IL12 Controls Triple-Negative Breast Cancer Growth and Metastasis

**DOI:** 10.3389/fonc.2020.00384

**Published:** 2020-03-24

**Authors:** Shanawaz M. Ghouse, Hong-My Nguyen, Praveen K. Bommareddy, Kirsten Guz-Montgomery, Dipongkor Saha

**Affiliations:** ^1^Department of Immunotherapeutics and Biotechnology, School of Pharmacy, Texas Tech University Health Sciences Center, Abilene, TX, United States; ^2^School of Graduate Studies, Rutgers University, New Brunswick, NJ, United States

**Keywords:** herpes simplex virus, oncolytic immunotherapy, breast cancer, metastasis, anti-angiogenesis

## Abstract

Triple-negative breast cancer (TNBC) is a difficult-to-treat disease with high rates of local recurrence, distant metastasis, and poor overall survival with existing therapies. Thus, there is an unmet medical need to develop new treatment regimen(s) for TNBC patients. An oncolytic herpes simplex virus encoding a master anti-tumor cytokine, interleukin 12, (designated G47Δ-mIL12) selectively kills cancer cells while inducing anti-tumor immunity. G47Δ-mIL12 efficiently infected and killed murine (4T1 and EMT6) and human (HCC1806 and MDA-MB-468) mammary tumor cells *in vitro*. *In vivo* in the 4T1 syngeneic TNBC model, it significantly reduced primary tumor burden and metastasis, both at early and late stages of tumor development. The virus-induced local and abscopal effects were confirmed by significantly increased infiltration of CD45^+^ leukocytes and CD8^+^ T cells, and reduction of granulocytic and monocytic MDSCs in tumors, both treated and untreated contralateral, and in the spleen. Significant trafficking of dendritic cells (DCs) were only observed in spleens of virus-treatment group, indicating that DCs are primed and activated in the tumor-microenvironment following virotherapy, and trafficked to lymphoid organs for activation of immune cells, such as CD8^+^ T cells. DC priming/activation could be associated with virally enhanced expression of several antigen processing/presentation genes in the tumor microenvironment, as confirmed by NanoString gene expression analysis. Besides DC activation/priming, G47Δ-mIL12 treatment led to up-regulation of CD8^+^ T cell activation markers in the tumor microenvironment and inhibition of tumor angiogenesis. The anti-tumor effects of G47Δ-mIL12 treatment were CD8-dependent. These studies illustrate the ability of G47Δ-mIL12 to immunotherapeutically treat TNBC.

## Introduction

Breast cancer is one of the most prevalent malignancies and the second most common cause of death among women in the United States (1). Almost 15 to 20% of breast cancer cases are classified as triple negative breast cancer (TNBC) variants, which lack the expression of estrogen and progesterone receptors, and human epidermal growth factor receptor 2 (HER2) proteins on tumor cells (2). Tumor recurrence, metastasis to other vital organs, and high heterogeneity are considered hallmarks of TNBC variants (3). Since TNBC does not express hormone receptors, the current treatment for TNBC mainly relies on surgery and chemotherapy; targeted therapy, such as hormone-based treatments or HER2 antagonists, is not a treatment option (4). Moreover, TNBC initially responds well but often develops resistance against chemotherapy (5), which underscores the need for developing novel therapeutic modalities that efficiently cure TNBC in patients.

Oncolytic viruses (OVs) (6), such as oncolytic herpes simplex viruses (OHSVs) are a promising therapeutic strategy for cancer treatment (7, 8). OHSVs are defined as genetically modified OVs that preferentially replicate in and kill tumor cells without harming normal cells (6). They have been established as strong *in situ* anticancer vaccines that activate antigen presenting cells (APCs), enhance APC-mediated tumor cell phagocytosis, augment antigen processing and presentation, and prime T cell responses (9). OHSVs have been successfully transitioned into clinical trials against various human cancers, including melanoma, glioma, pancreatic, and breast cancers (7, 8). In 2015, the U.S. Food and Drug Administration (FDA) approved the first OHSV (designated T-VEC) for the treatment of advanced melanoma in the United States. T-VEC is a genetically engineered OHSV expressing human granulocyte-macrophage colony-stimulating factor (hGM-CSF) (10), and is the furthest along in the clinic for cancer treatment (10). The safety and efficacy of T-VEC (as a monotherapy or combination therapy with paclitaxel) in TNBC patients is under clinical trial evaluation (8, 11, 12). However, T-VEC has not demonstrated durable responses in a majority of advanced melanoma patients (10), especially those with visceral metastases (13), which raises questions about its possible long-term efficacy in TNBC patients with metastatic disease.

G47Δ-mIL12 (14) is a genetically engineered OHSV that has similar genetic modifications to T-VEC (15, 16) but contains an extra safety feature [i.e., ICP6 inactivation that restricts OHSV replication to cancer cells (16)] and expresses murine Interleukin 12 (IL-12) (instead of GM-CSF). Upon infection of tumor cells, G47Δ-mIL12 releases a significant amount of IL12 (14), a master regulator of antitumor immunity, that enhances activation of dendritic cells and T lymphocytes, induces IFN-γ production, and inhibits angiogenesis (17–19). Previous reports affirm G47Δ-mIL12 as a potent oncolytic viral therapy for glioblastoma (14) and malignant peripheral nerve sheath tumors (20).

In this study, we have chosen to evaluate the therapeutic efficacy of G47Δ-mIL12 in a 4T1 tumor model, which is an immune-competent, highly tumorigenic, and invasive mouse mammary carcinoma that can spontaneously metastasize from the primary tumor in the mammary gland to multiple distant sites, such as lung (21). In addition, 4T1 serves as a model for stage IV of advanced breast cancer in humans. We found that G47Δ-mIL12 efficiently infected and eliminated both murine and human TNBC cells *in vitro*. *In vivo*, G47Δ-mIL12 treatment effectively inhibited 4T1 tumor growth, both primary and contralateral, and prevented metastasis to the lungs, which were associated with an enhanced APC activation, increased intratumoral CD8^+^ T-cell infiltration with subsequent reduction in myeloid-derived suppressor cells (MDSCs), and inhibition of angiogenesis. G47Δ-mIL12 exerted its anti-TNBC effects in a CD8^+^ T cell-dependent manner. These studies establish G47Δ-mIL12 virus as a powerful oncolytic immunotherapeutic agent for TNBC.

## Materials and Methods

### Cells and Viruses

Mouse (4T1 and EMT6) and human (HCC1806 and MDA-MB-468) mammary tumor cells were purchased from American Type Culture Collection (ATCC) and grown in Dulbecco's Modified Eagle Medium (DMEM) (ThermoFisher Scientific) supplemented with 10% heat-inactivated fetal bovine serum (HyClone), 2 mM L-glutamine (Corning), 1% MEM non-essential amino acids (Gibco), 1% sodium pyruvate (Gibco), and 0.5% penicillin G-streptomycin sulfate-amphotericin B complex (Corning). Cells were trypsinized with 0.05% trypsin supplemented with 0.54 mM EDTA (Corning) for passaging. Cells were low-passage and confirmed to be mycoplasma-free (LookOut mycoplasma kit, Sigma).

G47Δ-mIL12, an oHSV encoding IL-12, was constructed from G47Δ [containing deletions in α47 and γ34.5 genes and an inactivating insertion of *Escherichia Coli* LacZ into ICP6 (22)] by insertion of mouse IL-12 cDNA (p35 and p40 units are separated by two bovine elastin motifs) into the ICP6 gene (14). G47Δ-mCherry was described previously (14). Prior to *in vitro* and *in vivo* studies, the titers of infectious G47Δ-mIL12 virus were determined by plaque assay on Vero cells (14).

### Mice

Female BALB/c mice (aged 8–9 weeks) were obtained from the Jackson laboratory (Bar Harbor, ME) and utilized for all *in vivo* mouse studies involving the 4T1 mammary tumor cell line (21). Mice were housed at the Texas Tech University Health Sciences Center (TTUHSC) Laboratory *Animal* Resources Center (LARC)-Abilene under BSL2 conditions. All mouse procedures were approved by the Institutional Animal Care and Use Committee (IACUC) at the TTUHSC.

### Cell Viability Assay

Mouse and human mammary tumor cells were dissociated and seeded into 96-well plates (3,000 cells per well for mouse lines and 10,000 cells per well for human lines), treated with G47Δ-mIL12 at the indicated multiplicity of infection (MOI), incubated at 37°C for up to 72–96 h and CellTiter96 AQueous One Solution Cell Viability (MTS) Assays (Promega) performed according to the manufacturer's instructions. Values for virus-infected cells were normalized to those for mock-infected cells (percent cell viability). The experiments were performed in triplicate and repeated at least 2–4 times. Dose response curves and IC50 values were calculated using Prism 7 GraphPad software version 7.0e.

### Tumor Immunotherapy With G47Δ-mIL12 at Early-Stages of Tumor Development

Mice were implanted subcutaneously (s.c.) with 1 × 10^5^ 4T1 tumor cells into the mammary fat pad to generate orthotopic breast tumors. When tumors were palpable and reached 50-70 mm^3^ in tumor volume, mice were randomly divided into groups and intratumorally (i.t.) treated with G47Δ-mIL12 (in 25 μl PBS) or PBS on days 6, 9, 12, 15, and 18 post-tumor implantations. Tumors were measured at regular intervals with a digital caliper throughout the course of the experiment. The tumor volume was calculated using the following formula: (length x width x depth)/2.

### Tumor Immunotherapy With G47Δ-mIL12 at Late-Stages of Tumor Development

Orthotopic 4T1 tumors were established bilaterally in the right and left axillary mammary fat pads (1 × 10^5^ cells per mammary pad) on day 0. When tumors reached between 100–125 mm^3^ in volume, tumors located at the right axillary mammary fat pad were intratumorally injected with PBS or G47Δ-mIL12 on days 10, 13, 16, and 19, while the tumors at the left axillary mammary fat pad remained untreated and served as contralateral tumors. Tumor volumes (treated and untreated) were periodically measured by caliper, and mice were followed for survival until they become moribund or tumors reached to their burden limit, i.e., 1.5 cm in size.

### Metastatic Tumor Study

4T1 tumor cells (1 × 10^5^) were implanted s.c. into the mammary fat pad and treated i.t. with G47Δ-mIL12 or PBS as described above in “Tumor immunotherapy with G47Δ-mIL12 at early-stages of tumor development” section. Mice were sacrificed on day 21 post-tumor implantation, and lungs were isolated and fixed in Bouin's fixative solution (23). Twenty-four hours post-fixation, Bouin's solution was replaced by 70% ethanol and the number of lung surface metastatic nodules were counted with a Nikon Stereo Microscope with Plan APO 1x WD70 objective (23). Images for lung surface metastatic nodules were captured by Motic FEIN OPTIC SMZ-168 stereo microscope connected with a ToupTek digital camera.

### Immune Cell Depletion Studies

4T1 tumor cells (1 × 10^5^) were implanted s.c. into the mammary fat pad and treated with G47Δ-mIL12 or PBS injected i.t. on days 8, 11, 14, and 17 post-tumor implantations. For CD8^+^ cell depletion, mice were injected intraperitoneally (i.p.) with anti-CD8a antibodies (clone 2.43, 10 mg/kg, BioXCell) (24) or isotype control rat IgG2b antibodies specific to keyhole limpet hemocyanin (clone LTF-2, 10 mg/kg, BioXCell) (24) on days−4 and−1 prior to tumor implantation and on days 4, 8, 12, and 16 post-tumor implantation (25). Three groups of mice were included in CD8^+^ immune cell depletion studies: group 1 received PBS + IgG2b, group 2 received G47Δ-mIL12 + IgG2b, and group 3 received G47Δ-mIL12 + anti-CD8a. The concurrent tumor load study was performed as described above in this methods section. Mice were monitored for ill health and euthanized before becoming moribund. Tumor free survival curves were generated for the day each mouse reached its tumor burden limits, i.e., a maximum diameter of 15 mm.

### Multi-Color Flow Cytometry

For multi-color flow cytometric analysis (FACS), mammary tumor tissues were harvested and minced, and single-cell suspensions prepared by incubation of minced tissues in RPMI 1640 medium containing 10 mg/mL Collagenase (Roche), 0.4mg/mL DNase I (Roche), and 100 μg/mL Trypsin inhibitor (Sigma) for 30 min at 37°C. Enzymatic digestion was stopped by adding RPMI containing 10% fetal bovine serum (FBS; Corning), triturated, passed through a 70-μm cell strainer, washed twice with PBS, resuspended in FACS buffer, and counted using Vi-CELL XR Cell Viability Analyzer (Beckman Coulter). The samples were pre-incubated with purified anti-CD16/32 unconjugated antibody (clone 93) to block Fc receptors prior to surface staining with fluorochrome-conjugated anti-mouse monoclonal antibodies, which include: Brilliant Violet 605 anti-mouse CD45 (clone 30-F11), Alexa Fluor 700 anti-mouse CD3 (clone 17A2), PerCP/Cyanine5.5 anti-mouse CD4 (clone GK1.5), PE/Cy7 anti-mouse CD8a (clone 53-6.7), PE anti-mouse CD86 (clone GL-1), PerCP/Cyanine5.5 anti-mouse F4/80 (clone BM8), APC/Cyanine7 anti-mouse CD11c (clone N418), Alexa Fluor 700 anti-mouse/human CD11b (clone M1/70), Brilliant Violet 570 anti-mouse Ly6G (clone 1A8), PE/Cy7 anti-mouse Ly6C (clone HK1.4), APC anti-mouse CD274 (clone 10F.9G2), and Brilliant Violet 650 anti-mouse I-A/I-E (clone M5/114.15.2), as well as appropriate isotype control antibodies, as described (24, 26). All antibodies were purchased from Biolegend. Fixable Viability Dye eFluor 506 (eBioscience) was used to stain dead cells as per manufacturer instructions. Intracellular FOXP3 staining was performed using PE-conjugated anti-mouse FOXP3 antibody (clone MF-14, Biolegend) following the FOXP3 intracellular staining protocol (eBioscience). For multi-color FACS staining of spleens and tumor-draining lymph nodes, single cell suspensions from these organs were prepared as described (14) and stained as above. Fluorescent minus one (FMO) controls were included for each color-conjugated anti-mouse antibody, e.g., fluorescent minus F4/80 means staining cells with all colors except PerCP/Cyanine5.5 anti-mouse F4/80 (24). UltraComp eBeads (eBioscience) were used to prepare single-color compensation controls for each fluorescently conjugated antibody according to manufacturer instructions (26). Single-cell suspensions from harvested tissues were used to prepare a single-color compensation control for fixable viability dye eFluor 506. Data were acquired on BD Fortessa and analyzed with FlowJo software version 10.6.1 (Tree Star). Scientific personnel involved in acquiring and gating the data was blinded to the treatments.

### Indirect Immunofluorescence Staining

Mammary tumor tissues were harvested, snap-freezed in Tissue-Tek O.C.T. compound (Sakura), 5 μm cryostat sections prepared, fixed in methanol for 10 min at −20°C, sections dried at −20°C for 30 min followed by drying by hair dryer for 5 min at room temperature, and rehydrated in DPBS for 5 min. Rehydrated sections were blocked by incubation with 1% bovine serum albumin (BSA) diluted in DPBS for 30 min at room temperature. Sections were then incubated overnight with a purified rat anti-mouse CD31 antibody (clone MEC 13.3, BD Pharmingen; 1:50 dilution in 0.1% BSA/DPBS) at 4°C in a humidified chamber. Following three washes in DPBS+0.1% Tween 20 (5 min each), sections were incubated with goat anti-Rat IgG (H+L) cross-adsorbed secondary antibody conjugated with alexa fluor 488 (Invitrogen; 1:200 dilution in 0.1% BSA/DPBS) for 45 min at room temperature protected from light, followed by 3 times wash with DPBS+0.1% Tween 20, and nuclear counterstained with Fluoro-Gel II with DAPI (Electron Microscopy Sciences). Sections (3–5 random fields/tumor section, *n* = 4 or 5 mice/group) were imaged at 20x magnification with a Nikon fluorescent microscope. ImageJ software (NIH) was used to quantify the CD31^+^ areas. Scientific personnel involved in acquiring fluorescent images and ImageJ analysis was blinded to the treatments.

### Nano String Gene Expression Analysis

BALB/c mice were bilaterally implanted with 4T1 tumor cells (1 × 10^5^) in left and right axillary mammary fat pads. Tumors (~125 mm^3^) in the right fat pad were treated with PBS or G47Δ-mIL12 on indicated days, whereas tumors in the left mammary pads were left untreated. Tumor tissues were harvested at indicated time points and gene expression analysis performed using the NanoString PanCancer Immune Profiling Panel as previously described (27). In brief, 100 ng of total RNA per sample was mixed with a 3′-biotinylated capture probe and a 5′-reporter probe tagged with a fluorescent barcode from the custom gene expression code set. Probes and target transcripts were hybridized at 65°C for 16 h. Hybridized samples were run on the prep station platform as recommended by the manufacturer's protocol. The samples were scanned at maximum scan resolution on the nCounter Digital Analyzer. Data were processed using nSolver Analysis Software and the nCounter Advanced Analysis module. For gene expression analysis, data were normalized using the geometric mean of housekeeping genes selected by the GeNorm algorithm. Gene expression signatures were analyzed using Nsolver advanced analysis software (4.0) according to the manufacturer's guidelines. For heatmap generation, normalized data were scaled, and average linkage performed using cluster 3.0, and heat maps were generated using JavaTree.

### Statistical Analysis

All statistical analysis was done with Prism 7 GraphPad software version 7.0e. To compare tumor growth kinetics, unpaired 2-tailed student *t*-test was performed on mean tumor volumes at indicated time points. For comparison of immune cells infiltrate data, unpaired student *t*-test was applied. Survival data were analyzed by Kaplan-Meier survival curves and comparisons were performed by Log Rank test. *P* values of < 0.05 were considered statistically significant.

## Results

### G47Δ-mIL12 Efficiently Kills Murine and Human Breast Cancer Cells

**Figure 1:**
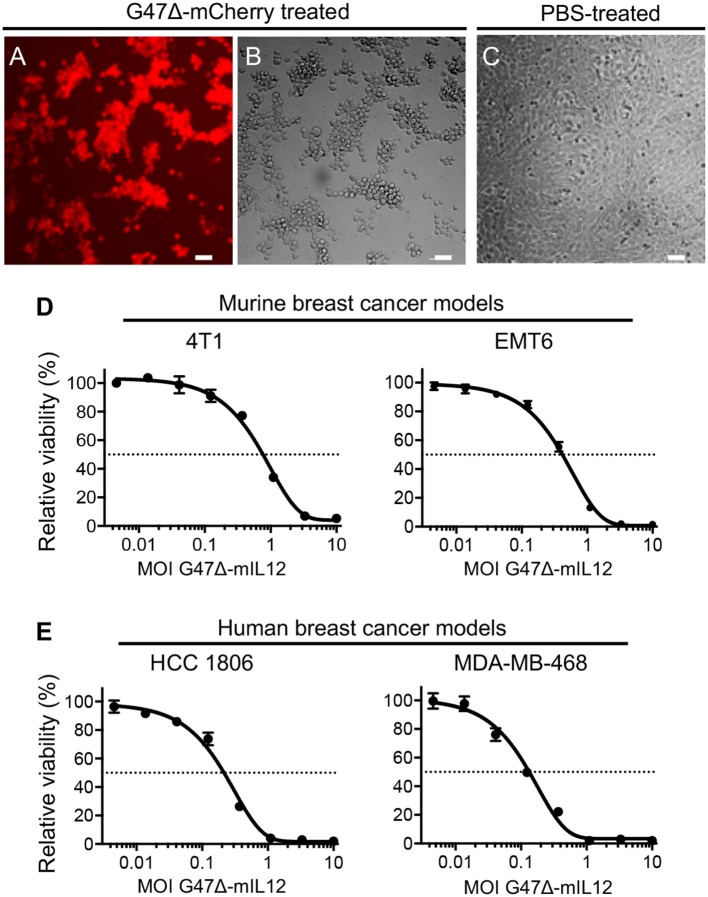
**(A–D)** G47Δ-mIL12 efficiently infects and kills murine breast cancer[[Inline Image]] cells. **(A–C)** 4T1 murine TNBC cells treated with G47Δ-mCherry (MOI = 1.0) or PBS and imaged at 24 hours post-treatment. mCherry red fluorescence image (in **A**) shows virus infection and phase contrast image (in **B**) shows round cytopathic cells following virus treatment. PBS treated 4T1 tumor cells were served as controls (in **C**). **(D)** Dose-response curves of G47Δ-mIL12 in 4T1 (left panel) and EMT6 (right panel) murine breast cancer models at 3 and 4 days post-treatment, respectively, as measured by MTS assay (Promega). **(E)** G47Δ-mIL12 efficiently kills human TNBC cells. Dose-response curve of G47Δ-mIL12 in HCC1806 (left panel) and MDA-MB-468 (right panel) TNBC cells at 4 days post-treatment, as measured by MTS assay. Mean ± SEM. Each graph represents an average of 2–4 experiments performed in triplicate.

*In vitro*, the G47Δ virus (our base OHSV with no IL-12 expression) efficiently kills human breast cancer cells with no observable cytotoxicity in normal breast cells even after 5 days of infection (28). It was previously demonstrated that that G47Δ-mIL12 (OHSV with IL12 expression) efficiently infects and kills syngeneic mouse brain tumor cells (14, 29) while releasing IL-12 in culture supernatants (14). Here, we tested the entry and sensitivity of 4T1 mouse mammary carcinoma cells to G47Δ-mCherry (an OHSV with fluorescent reporter mCherry expression) and G47Δ-mIL12, respectively. G47Δ-mCherry efficiently enters into 4T1 cells (Figure 1A) and induces cytopathic effects 24 hours after virus treatment (Figure 1B), not seen in untreated 4T1 cells (Figure 1C). G47Δ-mIL12 efficiently kills 4T1 murine TNBC cells (Figure 1D) with an IC50 = MOI ~ 0.8, which is comparable to our previous cytotoxicity studies in syngeneic mouse glioblastoma (GBM) models (14, 29). The cytotoxic activity of G47Δ-mIL12 (IC50 ~ 0.5) was confirmed in a second murine breast cancer model, EMT6 (Figure 1D). Similar to murine TNBC cells, the cytotoxic activity of G47Δ-mIL12 treatment is also efficient in human TNBC cells with IC50s of 0.15-0.25 (Figure 1E). These studies show that G47Δ-mIL12 can exhibit oncolytic effect both in mouse and human TNBC cells *in vitro*.

### G47Δ-mIL12 Treatment Controls TNBC Growth and Inhibits Metastasis

**Figure 2:**
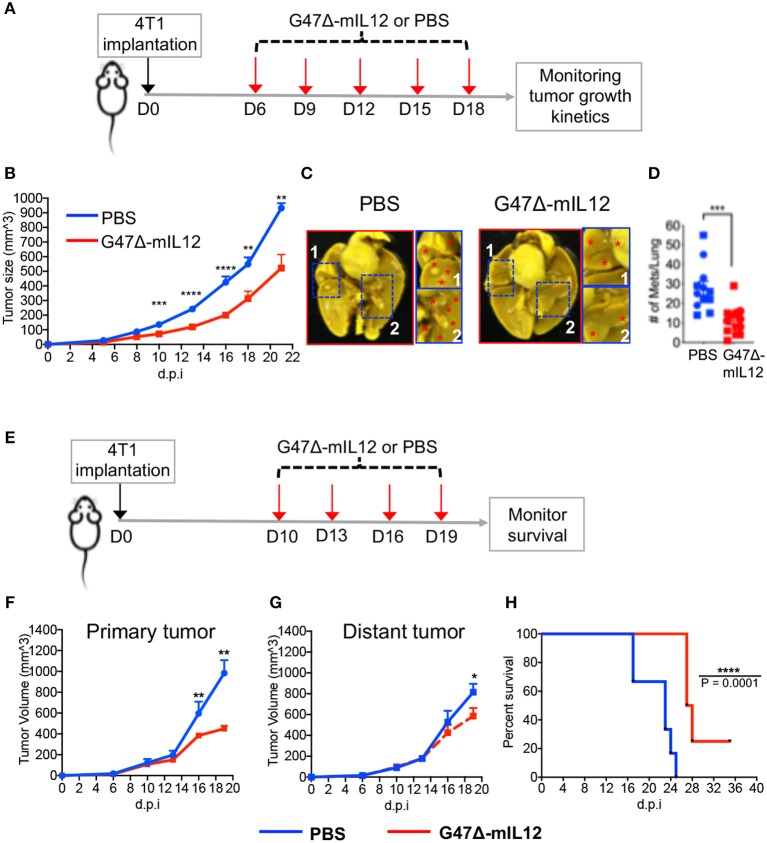
**(A-D)** G47Δ-mIL12 controls primary TNBC growth and inhibits metastasis in an orthotopic 4T1 mouse mammary carcinoma model. **(A)** Experimental schema. 1 × 10^5^ viable 4T1 cells in 100 μl PBS were injected into the right axillary mammary fat pad of female BALB/c mice. G47Δ-mIL12 (2 × 10^6^ PFU diluted in 25 μl PBS) or PBS injected intratumorally on day 6 (when tumors reached ~70 mm^3^), 9, 12, 15, and 18. Tumor volumes were measured every 2–3 days. **(B)** Tumor growth kinetics. Mean tumor volume of PBS injected tumors was compared to mean tumor volume of G47Δ-mIL12 injected tumors from day 10 to day 21. **(C)** Representative images of lungs. Mice from experiment 2B were euthanized on day 21 and lungs were fixed in Bouin's fixative and imaged at 24 hours post-fixation. Red asterisk indicates metastatic nodules at higher magnification in insets. **(D)** Total number of lung surface metastatic nodules counted with a stereo microscope. Mice from experiment 2B. Data presented by combining two independent experiments (*n* = 12 mice/group) and each experimental data are indicated by round- and square-shaped symbols. Mean ± SEM. Unpaired Student's *t*-test (two-tailed), ^**^P ≤ 0.01, ^***^P ≤ 0.001, ^****^P ≤ 0.0001; d.p.i., days post-tumor implantation. **(E-H)** G47Δ-mIL12 treatment controls growth of treated and untreated contralateral tumors at late-stage of tumor development and extends survival. **(E)** Experimental schema. Orthotopic 4T1 tumors were established bilaterally in the right and left axillary mammary fat pads (1 × 10^5^ cells per mammary pad) on day 0. When tumors reached between 100 and 125 mm^3^ in volume, tumors located at the right axillary mammary fat pad intratumorally injected with PBS (*n* = 6) or G47Δ-mIL12 (10^6^ pfu/injection) (*n* = 8) on days 10, 13, 16, and 19, and the tumors at the left axillary mammary fat pad remained untreated and served as contralateral tumors. Tumor volumes periodically measured by caliper and mice were followed for survival until they become moribund or until tumors reached to their burden limit, i.e., 1.5 cm in size. **(F, G)** Tumor growth kinetics of treated and untreated contralateral tumors. Mean tumor volume of PBS treatment group was compared to mean tumor volume of G47Δ-mIL12 treatment group on day 16 and 19 by Unpaired Student's *t*-test (two-tailed). ^*^P ≤ 0.05, ^**^P ≤ 0.01. **(H)** Kaplan-Meier survival curve. Median survival of mice treated with G47Δ-mIL12 (27.5 days; 25% of mice surviving until the end of experiment on day 36) was compared to median survival of mice treated with PBS (23 days; *P* = 0.0001) by Log Rank test. ^****^P ≤ 0.0001. d.p.i., days post-tumor implantation.

To test the therapeutic effects of G47Δ-mIL12 on tumor burden and metastasis, 4T1 TNBC cells (1 × 10^5^) were implanted subcutaneously into the mammary fat pad of BALB/c mice. When tumors reached ~70 mm^3^ in size, mice were treated with intratumoral injections of PBS or G47Δ-mIL12 (2 x 10^6^ PFU) on days 6, 9, 12, 15, and 18 (see schema in Figure 2A). G47Δ-mIL12 therapy significantly controlled the tumor growth compared to controls (*P* = 0.0003 at day 10, *P* ≤ 0.0001 at day 13, *P* ≤ 0.0001 at day 16, *P* = 0.0045 at day 18, *P* = 0.0082 at day 21; Figure 2B). Because metastasis is associated with a poor survival outcome in patients with TNBC (30), we ought to determine whether G47Δ-mIL12 treatment can inhibit metastasis. Three days after the last treatment (i.e., day 21), lungs were fixed in Bouin's fixative (23), and the number of metastatic nodules counted with a stereomicroscope (23). We observed that G47Δ-mIL12 oncolytic virus therapy significantly inhibited the metastatic ability of 4T1 tumor cells, as demonstrated by an almost 3-fold reduction in surface metastatic colonies in lungs (*P* = 0.0003 vs. PBS control; Figures 2C,D). Overall, these studies illustrate the ability of G47Δ-mIL12 virus therapy to effectively control primary and metastatic diseases associated with TNBC.

To determine anti-cancer effects of oncolytic virotherapy at late stages of tumor development, 4T1 tumor cells were implanted bilaterally in the mammary fat pad. When tumors reached ~100–125 mm^3^ in size, tumors on the right axillary mammary fat pad were treated with PBS or G47Δ-mIL12 on indicate days, while the tumors at the left axillary mammary fat pad remained untreated and served as contralateral tumors (Figure 2E). We observed that G47Δ-mIL12 treatment effectively and significantly controlled the growth of both treated and untreated tumors at late-stages of tumor development (Figures 2F,G), leading to significant extension of survival with 25% mice surviving long-term compared to PBS treatment group (Figure 2H).

### G47Δ-mIL12 Induces Local and Abscopal Immune Effects

**Figure 3:**
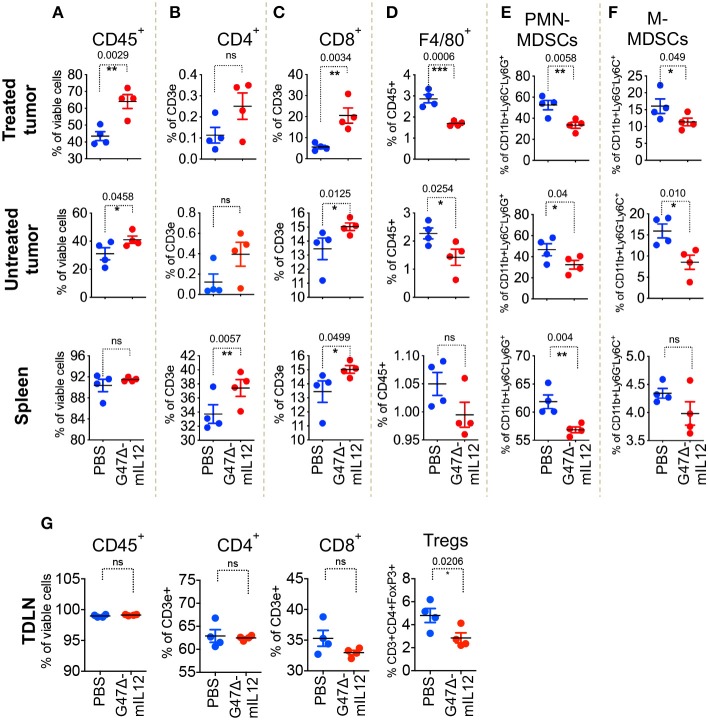
G47Δ-mIL12 induces local and abscopal immune responses. Orthotopic 4T1 tumors were established bilaterally in the right and left axillary mammary fat pads (1 × 10^5^ cells per mammary pad) on day 0. When tumors reached 100–125 mm^3^ in volume, tumors located at the right axillary mammary fat pad intratumorally treated with PBS or G47Δ-mIL12 (10^6^ pfu/injection) on days 10, 13, 16, and 19, and the tumors at the left axillary mammary fat pad remained untreated (*n* = 4 mice/group). On day 21, primary treated tumors, contralateral tumors, spleens and tumor draining lymph nodes (TDLNs) were harvested and subjected to multicolor flow cytometry as described in section Materials and Methods. Tumor infiltrating immune cells were gated based on gating strategy as presented in Supplementary Figure 1. **(A–F)** Frequencies of live CD45^+^ cells **(A)**, live CD3e^+^CD4^+^ T cells **(B)**, live CD3e^+^CD8a^+^ T cells **(C)**, live CD45^+^F4/80+ macrophages **(D)**, live polymorphonuclear MDSCs (CD11b^+^Ly6C^−^Ly6G^+^) **(E)**, and mononuclear MDSCs (CD11b^+^Ly6G^−^Ly6C^+^) **(F)** in treated tumors (panels in upper row), contralateral tumors (panels in 2^nd^ row), and spleens (panels in 3^rd^ row). **(G)** Frequencies of live CD45^+^ cells, live CD3e^+^CD4^+^ T cells, live CD3e^+^CD8a^+^ T cells, and CD3e^+^CD4^+^FoxP3^+^ regulatory T cells (Tregs) in tumor draining lymph nodes (TDLNs; panels in bottom row). Mean ± SEM. Statistically significant differences between groups are reported as *P*-values in the figures. Statistical significance was assessed by Student's *t* test. ^*^P ≤ 0.05, ^**^P ≤ 0.01, ^***^P ≤ 0.001; ns, not significant.

**Figure 4:**
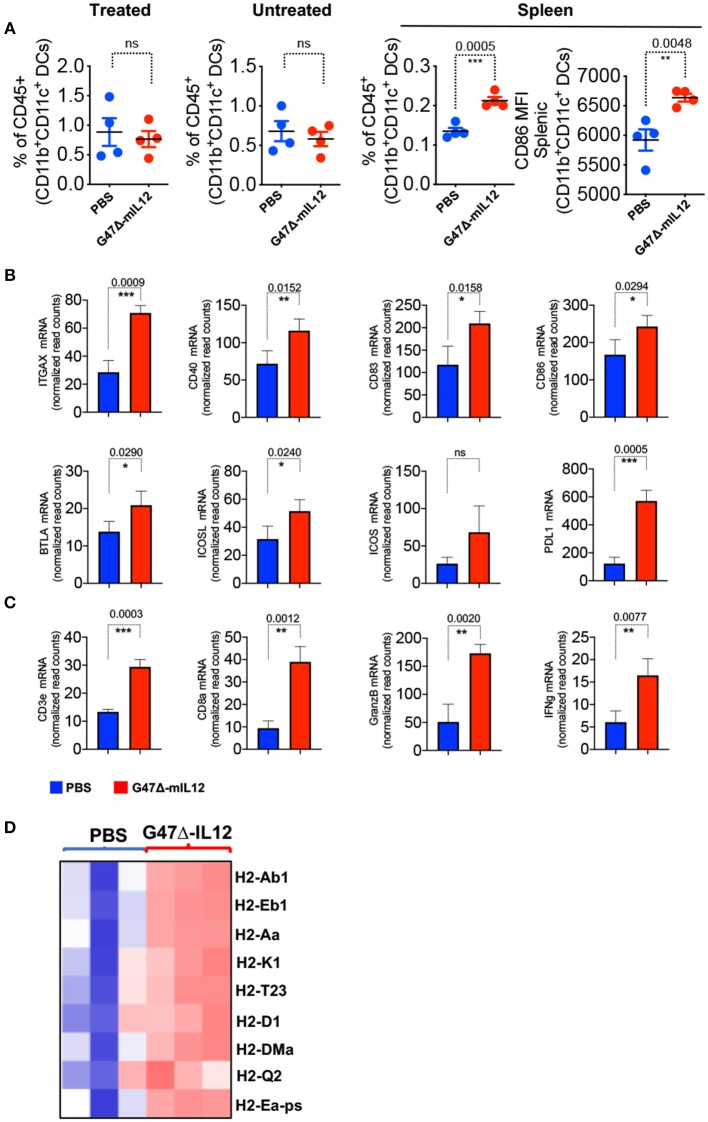
G47Δ-mIL12 treatment leads to DC maturation and T cell activation *in vivo*. **(A)** Frequencies of CD45^+^CD11b^+^CD11C^+^ DCs in treated, untreated contralateral tumors and spleen (left panel), and mean fluorescent intensity (MFI) of activated DCs (CD11b^+^CD11c^+^CD86^+^) in spleen (right panel). Same experiment as in Figure 3 (*n* = 4/group). Mean ± SEM. Statistically significant differences between groups are reported as *P*-values in the figures. Unpaired Student's *t* test (two-tailed), ^**^P < 0.01, ^***^P < 0.001; ns, not significant. **(B–D)** BALB/c female mice were implanted with 4T1 tumor cells (1 × 10^5^ cells) in the right axillary mammary fat pad and treated with PBS (*n* = 3) and G47Δ-mIL12 (*n* = 3) (10^6^ pfu/injection) on days 10, 13, 16, and 19 (as in Figure 3). Primary tumor tissues were harvested on day 21, RNA isolated, and Nano String gene expression analysis performed using the PanCancer Immune Profiling Panel kit as described in section Materials and Methods. mRNA levels of genes associated with DC maturation (*ITGAX, CD40)* and DC-specific co-stimulatory signaling *(CD83, CD86, BTLA, ICOS, ICOSL*) are presented in B, genes associated with CD8^+^ T cell activation (*CD3e, CD8*α*, Granzyme B*, and *IFN*-γ) are presented in **(C)**, and genes involved in antigen processing and presentation are presented as a heat map in D. Mean ± SEM. Statistically significant differences between groups are reported as *P*-values in the figures. Statistical significance was assessed by Unpaired Student's *t* test (two-tailed). ^*^P ≤ 0.05, ^**^P ≤ 0.01, ^***^P ≤ 0.001; ns, not significant.

To understand the role of different immune cell populations contributing to G47Δ-mIL12-mediated control of tumor growth and metastasis, orthotopic 4T1 tumors were established bilaterally in right and left axillary mammary fat pads of BALB/c mice. Due to the sample's scarcity for downstream applications, inoculated tumors were allowed to grow between 100 and 125 mm^3^. Tumors located at the right axillary mammary fat pad were treated with intratumoral injections of PBS or G47Δ-mIL12 on days 10, 13, 16, and 19, whereas tumors at the left axillary mammary fat pad remained untreated. To evaluate virus-induced abscopal effects and immune responses, treated tumors, contralateral tumors, and the spleens were harvested on day 21 and subjected to multicolor flow cytometry for immune cell analysis. First, we selected the CD45 surface marker to distinguish CD45- tumor cells from CD45^+^ hematopoietic immune cells by adopting a specific gating strategy (Supplementary Figure 1). G47Δ-mIL12 treatment resulted in following immune cell alterations in both local (treated) and contralateral (untreated) tumors and spleens that included: (1) significantly increased infiltration of CD45^+^ immune cells in treated and contralateral tumors (*P* ≤ 0.05 vs PBS; Figure 3A), (2) further characterization of CD45^+^ cells revealing a significant increase in infiltration of CD8^+^ T lymphocytes, but not CD4^+^ T cells (except spleen), in both tumor lesions and spleens (*P* ≤ 0.05 vs. PBS; Figures 3B,C), (3) significantly reduced macrophages and both granulocytic and monocytic MDSCs in both tumor lesions (Figures 3D–F), and (4) significant reduction of regulatory T cells (CD4^+^FoxP3^+^) observed in tumor draining lymph nodes from G47Δ-mIL12 treated mice (Figure 3G), which suggests G47Δ-mIL12-induced beneficial systemic immune responses. As expected, we have observed a significantly higher infiltration of CD45^+^ cells in virus-treated tumors as compared to untreated contralateral tumors, while no significant differences were observed in other immune cell population between virus treated and untreated tumors (Supplementary Figure 2). While CD8^+^ T cells are an important contributor in inducing effector anti-tumor immunity, MDSCs can inhibit adaptive anti-tumor immunity and are an obstacle to cancer immunotherapies (31). Thus, enhanced CD8^+^ T cell infiltration and reduced MDSC populations in treated and untreated tumor lesions due to virus treatment clearly show the ability of G47Δ-mIL12 to induce beneficial anti-tumor immune effects (local and abscopal), which could play a critical role in inhibiting primary and metastatic TNBC (Figure 2).

### G47Δ-mIL12 Treatment Leads to DC Maturation and T Cell Activation *in vivo*

OVs have been established as strong *in situ* anti-cancer vaccines (9, 14, 24, 29, 32) that activate antigen presenting cells (APCs), augment antigen processing and presentation, and prime CD8^+^ T cell responses (9). We observed no significant changes in dendritic cell (DC) population in virus-treated tumor lesions (Figure 4A). However, G47Δ-mIL12 treatment led to significant trafficking of DCs in the spleens of treated mice versus PBS control group (Figure 4A), indicating that DCs primed in the tumor microenvironment following virus treatment likely trafficked to lymphoid organs for antigen presentation to immune cells such as CD8^+^ T cells (Figure 3C). Spleen-localized DCs were overwhelmingly in a mature APC state based on significant expression of the activation marker CD86 in virus-treated group versus the PBS control group (Figure 4A), which is critical for T cell activation (33). Nano String gene expression analysis further confirmed upregulation of genes involved in DC maturation (e.g., *ITGAX, CD40)*, DC-specific co-stimulatory signaling *(CD83, CD86, BTLA, ICOS, ICOSL*) and CD8^+^ T cell activation (*CD3e, CD8*α*, Granzyme B*, and *IFN*-γ) (34) (Figures 4B,C). Gene expression analysis also revealed enhanced expression of antigen processing/presentation genes (e.g., *H2-Ab1, H2-Eb1, H2-Aa, H2-k1, H2-T23, H2-D1, H2-DMa, H2-Q2, H2-Ea-ps*) in virus injected tumor lesions (Figure 4D), suggesting virus-induced DC priming and activation, and enhanced antigen presentation. Altogether, these data establish the ability of our G47Δ-mIL12 virus to induce efficient *in situ* vaccine effects in promoting DC maturation and antigen presentation that eventually could be responsible for promoting CD8^+^ T cell responses in virus-injected and non-injected tumor lesions.

### G47Δ-mIL12 Treatment Induces Anti-angiogenic Effects *in vivo*

**Figure 5:**
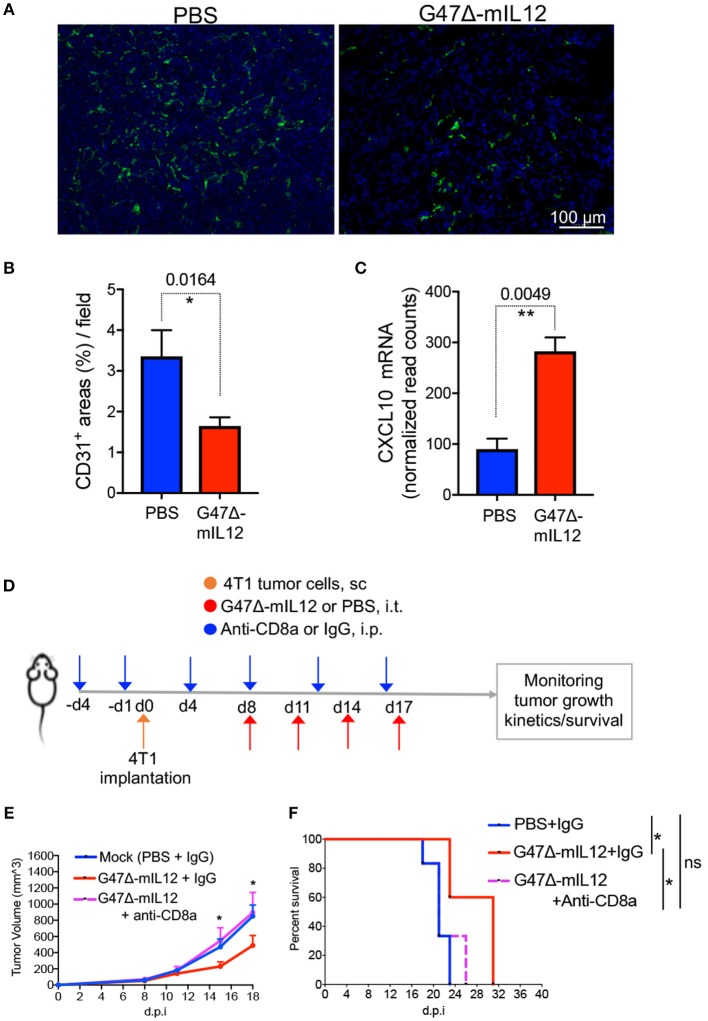
**(A-C)** G47Δ-mIL12 treatment induces anti-angiogenic effects. **(A)** Immunofluorescence staining of CD31^+^ tumor blood vessels after G47Δ-mIL12 treatment. Same experiment as Figure 2A. Representative images are presented; scale bar = 100 μm. **(B)** CD31^+^ positive areas from 3 to 5 random fields/tumor section (1 section/mouse; *n* = 5 for PBS and *n*= 4 mice/group for G47Δ-mIL12) were measured by ImageJ software and presented as Mean ± SEM. **(C)** G47Δ-mIL12 treatment increases expression of CXCL10 (IP10) in tumor microenvironment. Same as experiment 4B-D. Mean ± SEM. Unpaired Student's *t* test (two-tailed). ^*^P ≤ 0.05, ^**^P ≤ 0.01. **(D-F)** CD8a^+^ T cell depletion abrogates G47Δ-mIL12-induced anti-TNBC efficacy. **(D)** Experimental schema. BALB/c female mice were implanted with 4T1 tumor cells (1 × 10^5^ cells) in the mammary fat pad on day 0. When tumors reached approximately 80-90 mm^3^ in volume (day 8), mice were treated intratumorally with G47Δ-mIL12 (1 × 10^6^ pfu / 25 μl in PBS) or PBS on days 8, 11, 14, and 17 (upward red arrows). Anti-CD8a antibody (5 mg/kg) or isotype control IgG (5 mg/kg rat IgG) injected IP on day-4 and−1 prior to tumor implantation and on days 4, 8, 12, and 16 post-tumor implantations (downward blue arrows). **(E)** Growth kinetics of tumors in mice treated with PBS/IgG (*n* = 6), G47Δ-mIL12/IgG (*n* = 5), or G47Δ-mIL12/Anti-CD8a (*n* = 6). Mean tumor volume of PBS/IgG treated mice was compared to mean tumor volume of G47Δ-mIL12/IgG treated mice on indicated days. Mean ± SEM of all mice presented. Statistical differences between groups were compared by Unpaired 2-tailed Student's *t* test. ^*^P ≤ 0.05. **(F)** Kaplan-Meier survival curve. Mice from experiment **(E)** were followed for ill health and the survival curve was generated on the day each mouse reached its tumor burden limit, i.e., a maximum diameter of 15 mm. Median survival of mock (PBS+IgG) treated mice (21 days; *n* = 6) was compared to mice treated with G47Δ-mIL12+IgG (31 days; *n* = 5, *P* = 0.0116) and G47Δ-mIL12+anti-CD8a (21 days; *n* = 6, *P* = 0.4070). Median survival of mice treated with G47Δ-mIL12+IgG (31 days) was compared to mice treated with G47Δ-mIL12+anti-CD8a (21 days; *P* = 0.0369). The survival data was analyzed by Log Rank test. ^*^P ≤ 0.05; ns, not significant; s.c., subcutaneous; i.p., intraperitoneal; i.t., intratumoral; d.p.i., days post-tumor implantation.

Interleukin 12 (IL-12) is an anti-angiogenic cytokine (19). IL-12 elicits its anti-anti-angiogenic effects through release of IFN-γ, which activates IFN-inducible protein 10 [IP-10 or CXC chemokine ligand (CXCL) 10], a chemokine that mediates chemotaxis of lymphocytes and angiostatic effects (19, 35, 36). It was previously demonstrated that G47Δ-mIL12 treatment can inhibit angiogenesis (14). In order to determine whether viral expression of IL-12 (i.e., G47Δ-mIL12) can induce any anti-angiogenic effects in murine TNBC model, 4T1 TNBC cells (1 × 10^5^) were implanted subcutaneously into the mammary fat pad of BALB/c mice, and treated intratumorally with PBS or G47Δ-mIL12 (2 x 10^6^ PFU) (see schema in Figure 2A). Three days after the last treatment (i.e., day 21), methanol-fixed cryostat sections were subjected to indirect immunofluorescence staining for CD31^+^ tumor vasculatures, as described in ‘Materials and Methods’ section. We observed that G47Δ-mIL12 treatment significantly reduced CD31^+^ tumor vascularity by 2-fold compared to PBS treatment mice (Figures 5A,B). Nano String gene expression analysis also revealed significantly increased gene expression of anti-angiogenic molecule CXCL-10 (IP-10) in 4T1 tumors (*P* = 0.0049 vs. PBS) (Figure 5C). These studies demonstrate anti-angiogenic properties of the G47Δ-mIL12 virus in TNBC.

### CD8^+^ T Cell Depletion Abrogates G47Δ-mIL12-Induced Anti-TNBC Efficacy

It was previously demonstrated that G47Δ-mIL12 treatment leads to local production of IL-12 (14). Viral release of IL-12 is accompanied by a marked release of IFN-γ (14), which facilitates CD8^+^ T-cell-mediated killing of tumor cells (37). Because there is an increased intratumoral CD8^+^ T cell infiltration (Figure 3C) with enhanced cytotoxic T cell activation markers (Figure 4C) following G47Δ-mIL12 treatment, we hypothesized that anti-TNBC efficacy of G47Δ-mIL12 can be CD8^+^ T cell-dependent. To address whether the changes seen in tumor infiltrating CD8^+^ T immune cells are necessary to elicit anti-tumor efficacy of G47Δ-mIL12, we performed antibody depletion studies of CD8^+^ cells as described (25). CD8^+^ T cell depletion was confirmed by flow cytometric analysis (Supplementary Figure 3). When orthotopic mammary tumors reached 80-90 mm^3^ in volume, mice were treated intratumorally with G47Δ-mIL12 or PBS, and intraperitoneally with anti-CD8 antibodies or isotype control IgG on indicated days (Figure 5D). The results show that depletion of CD8^+^ cells abrogated anti-tumor effects of G47Δ-mIL12 treatment, as demonstrated by similar tumor growth kinetics and tumor free survival as mock-treated animals (Figures 5E,F). Overall, these studies demonstrate that G47Δ-mIL12-mediated anti-tumor immune response is CD8-dependent.

## Discussion

Current treatments for TNBC patients are limited to surgery and chemotherapy. TNBC patients initially respond well to chemotherapy but most patients develop resistance at advanced stages. Moreover, immune checkpoint blockade (ICB) immunotherapy alone (e.g., anti-PD-1), which is usually successful in immunologically “hot” tumors (e.g., subsets of melanoma) (38, 39), produces a low overall response rate (<5%) in TNBC patients (40), and a recent Phase III anti-PD-1 trial in TNBC did not meet its endpoint (KEYNOTE-119). In some patients, the resistance to chemo- or -ICB therapy can be overcome to some extent. For instance, a chemotherapeutic agent given in combination with an ICB (e.g., anti-PD-L1, designated atezolizumab; anti-PD-1, designated pembrolizumab) can lead to higher progression-free survival (IMpassion130) (41) and a more pathological complete response (pCR) rate (KEYNOTE-522) (42), respectively, compared to chemotherapy alone. However, a vast majority of patients (i.e., 79.4% in the intention-to-treat population) treated with the combination (ICB immunotherapy + chemotherapy) have experienced disease progression or died (IMpassion130) (41). Thus, there is an unmet medical need to develop new treatment regimen(s) that efficiently activate the immune system and eventually eradicate or control primary tumor growth and metastasis. An alternative and potentially improved immunotherapeutic approach involves application of oncolytic immunovirotherapy that has shown promise in various malignancies preclinically, including TNBC (7, 8). In this study, first we tested the ability of G47Δ-mIL12 to infect and kill mouse and human TNBC cells *in vitro*. Then we evaluated the therapeutic efficacy of G47Δ-mIL12 as a monotherapy in an immunocompetent, syngeneic, and highly metastatic 4T1 mouse model of mammary carcinoma. In this model, G47Δ-mIL12 monotherapy significantly inhibited TNBC tumor growth and prevented metastasis, both at early and late-stages of tumor development, and was associated with increased DC maturation and T cell activation, enhanced infiltration of CD8^+^ T cells and reduced infiltration of MDSCs into treated and distant tumors. The anti-tumor efficacy of G47Δ-mIL12 treatment on primary tumor growth was abrogated in the absence of CD8^+^ T cells.

There has not been any study performed so far testing the cytotoxic activity of OHSV in murine TNBC cell lines *in vitro*, other than a study testing viral replication in 4T1 tumor cells (43). Here, we observed that both 4T1 and EMT6 murine breast cancer cells are sensitive to G47Δ-mIL12 treatment with IC50s of MOIs 0.9 and 0.5, respectively, which are similar to the killing activity of G47Δ-mIL12 *in vitro* in syngeneic mouse glioblastoma cells (14, 29). Human cancer cells are typically more permissive, and therefore, should be more sensitive to OHSV treatment than mouse cancer cells (22). Indeed, the tumor cell killing efficiency of G47Δ-mIL12 is better in human TNBC cells (HCC1806 and MDA-MB-468), and requires a lesser MOI (IC50=0.15-0.25), which is at least 2-6 fold lower than that in mouse breast cancer models. Similar to G47Δ-mIL12, other OHSVs with different genetic backgrounds or modifications also efficiently kills human TNBC cells (44–46).

A recently published study in a pre-surgical neoadjuvant setting shows that an ICP0-deleted OHSV replicates poorly in the 4T1 model and is not effective at all in controlling the growth of the injected tumors in a subcutaneous 4T1 flank model, despite predominantly controlling the growth of secondary 4T1 tumors, which resembles TNBC metastasis (43). Similar to Martin et al. (43), the present study shows that G47Δ-mIL12 monotherapy significantly reduced TNBC metastasis, as demonstrated by a significant reduction of surface metastatic nodules in the lungs (Figures 2C,D), and significant growth inhibition of non-injected contralateral tumors (Figure 2G). Although we found similarity in controlling metastatic tumor burden, Martin et al. (43) findings contradict other key findings in our study. For instance, G47Δ-mIL12 efficiently replicated in and killed mouse cancer cells (14) (Figure 1), significantly controlled the growth of injected tumors (Figures 2B,F), and extended survival (Figure 2H), as opposed to poor viral replication and “no” anti-tumor efficacy against injected tumors observed by an ICP0-deleted OHSV (43). The contradictory observations can be explained by the fact that G47Δ-mIL12 has an intact ICP0, a critical immediate-early protein of viral tegument, which is freed into the cytosol upon infection to prepare the cell for virus replication (16). Intact ICP0 in the G47Δ-mIL12 virus may have played a role making G47Δ-mIL12 efficacious against TNBC models, both *in vitro* and *in vivo*.

Tumor microenvironment plays a vital role in the success of oncolytic virus therapy. Tumor infiltrating CD8^+^ T cells are associated with reduced recurrence and longer survival among TNBC patients (47, 48). In this study, the quantification of immune infiltrate demonstrated a significant increase in CD8^+^ T cells in both G47Δ-mIL12-treated and untreated contralateral tumors and spleens (Figure 3C). When CD8^+^ T cells were depleted, G47Δ-mIL12 treatment efficacy was abrogated (Figures 5E,F), indicating CD8^+^ T cells are essential for the antitumor effects of G47Δ-mIL12. Myeloid-derived suppressor cells (MDSCs) are immuno-suppressive cells that inhibit antitumor immunity (31). One of the striking findings in our study was a significant reduction of monocytic and granulocytic MDSCs in both treated and untreated tumors following oncolytic immunovirotherapy (Figures 3D,F). In contrast to MDSCs, we did not find any significant treatment effect on DC population in either treated or contralateral tumors. However, we observed a significant increase in splenic DCs, which were overwhelmingly positive for CD86, an activation marker for DCs (Figure 4A). It is important to note that IL-12 in G47Δ-mIL12 virus may have played a critical role in activating APCs, since IL-12 is a potent MDSC modulator and shifts splenic MDSCs (isolated from 4T1-tumor bearing BALB/c mice) into CD11c^+^CD86^+^ activated DCs (17). Nano String gene expression analysis further confirmed DC maturation and activation by demonstrating virally enhanced expression of genes associated with DC activation, antigen processing and presentation, and T cell activation in G47Δ-mIL12 treatment tumors (Figures 4B,D). Since G47Δ-mIL12 is an efficient modulator of antigen processing/presentation, it remains to be determined whether other APC activators can synergize with the *in situ* vaccine effects of G47Δ-mIL12 treatment and improve the therapeutic outcome in TNBC.

Recently published clinical studies in TNBC patients demonstrate that combination of chemotherapy with an ICB results in significantly higher progression-free survival or pCR rate in PD-L1-positive patient population compared to PD-L1-negative TNBC patients [KEYNOTE-522 (42) and IMpassion130 (41)]. This suggests that PD-L1 expression plays a key role in determining the treatment efficacy. An important limitation of the work presented here is that the G47Δ-mIL12 monotherapy did not eliminate 4T1 primary tumors and metastasis, and we observed that virus treatment dramatically upregulated PD-L1 expression in 4T1 tumors (Figure 4B). It was previously demonstrated that ICB treatment improves therapeutic outcome of OHSV therapy in mouse glioblastoma (24) and human advanced melanoma (49). Thus, future studies involving combination immunovirotherapy (i.e., G47Δ-mIL12 + ICB) can further augment the therapeutic effects of G47Δ-mIL12 and may lead to complete eradication of TNBCs.

Because IL-12 is well known for its anti-angiogenic properties (19), it was our expectation that G47Δ-IL12 treatment would lead to inhibition of tumor angiogenesis in TNBC model. Indeed, we observed a significant reduction of 4T1 tumor vascularity following G47Δ-IL12 treatment compared to PBS treatment group (Figures 5A,B). Production of CXCL-10 (IP-10) is inversely correlated with tumor growth and angiogenesis (50). Here, we noticed a significant upregulation of CXCL-10 in virus-treated 4T1 tumors (Figure 5C). These findings are similar to what was reported previously with G47Δ-IL12 in a mouse glioblastoma model (14), such as inhibition of glioblastoma angiogenesis with an increased expression of intratumoral CXCL-10 (14). These studies also indicate that anti-angiogenic effects of G47Δ-IL12 can synergize with other anti-angiogenic agents and may improve the therapeutic outcome (to be tested in future studies).

In summary, we show for the first time that an OHSV expressing mouse IL-12 (G47Δ-mIL12) virus effectively infects and kills mouse and human breast cancer cell lines. Treatment of syngeneic mice bearing 4T1 TNBC tumors with G47Δ-mIL12 lead to a CD8^+^ T cell-dependent inhibition of 4T1 tumor growth, inhibition of tumor angiogenesis, and prevention of lung metastasis, suggesting local and systemic anti-cancer effects of G47Δ-mIL12. Finally, G47Δ-mIL12 treatment also leads to an increase in PD-L1 expression in tumors, suggesting the therapeutic benefit of combining G47Δ-mIL12 with anti-PD-1/PD-L1 antibodies. Overall, these findings strongly suggest that G47Δ-mIL12-based immunovirotherapy could be a promising therapeutic approach for TNBC patients.

## Data Availability Statement

Publicly available datasets were analyzed in this study, these can be found in the NCBI Gene Expression Omnibus (GSE144333).

## Ethics Statement

The animal study was reviewed and approved by Institutional Animal Care and Use Committee (IACUC) at the TTUHSC.

## Author Contributions

SG performed experiments, prepared figures, wrote initial draft, and edited the manuscript. H-MN performed experiments, made figures, wrote, and edited the manuscript. PB performed Nano String experiments/analysis and edited the manuscript. KG-M helped with the animal experiments and analysed data, edited the manuscript. DS performed experiments, conceptualization, original draft preparation, preparation of figures, review and editing, data analysis, and funding acquisition. All authors agree to be accountable for the content of the work.

### Conflict of Interest

PB is an employee of Replimune Inc. The authors declare that the research was conducted in the absence of any commercial or financial relationships that could be construed as a potential conflict of interest.
