# Supplementary material for: Oncolytic Herpes Simplex Virus Encoding IL12 Controls Triple-Negative Breast Cancer Growth and Metastasis
Source: Front Oncol. 2020 Mar 24;10:384. doi: 10.3389/fonc.2020.00384 (PMC7105799; doi:10.3389/fonc.2020.00384)
Supplement: Supplementary file 1 [file Table_1.DOCX]

Supplementary Material

**Supplementary Figure 1. Gating strategy for flow cytometry analysis of infiltrating lymphoid and myeloid cells in tumor tissues, and tumor draining lymph nodes**. Related to Figure 3 and Figure 4A. **(A)** Gate for nucleated cells based on the size and scattering properties of the cells, FCS-A and SSC-A, respectively. **(B)** Nucleated cells were further plotted in FSC-A and FSC-H to gate single cells and exclude doublets. **(C)** From the single cell gate, viable cells were defined as cells negative for fixable viability dye (FVD). **(D)** CD45^+^ cells were gated to exclude the tumor cells and other non-immune cells. **(E)** A lymphocyte gate (CD45^+^CD3e^+^) was made on cells positive for both CD45 and CD3e. **(F)** T cells were further divided into CD4^+^, CD8a^+^, and CD4^−^CD8^−^ populations. **(G)** Dendritic cells were defined as cells double positive for CD11b and CD11c (CD11b^+^CD11c^+^). **(H)** Macrophages were defined as F4/80^+^ cells. **(I)** Granulocytic and monocytic MDSCs were defined based on Ly6G and Ly6C expression markers. **(J)** Regulatory T cells were defined as the CD45^+^CD4^+^ T cell subsets positive for FoxP3.


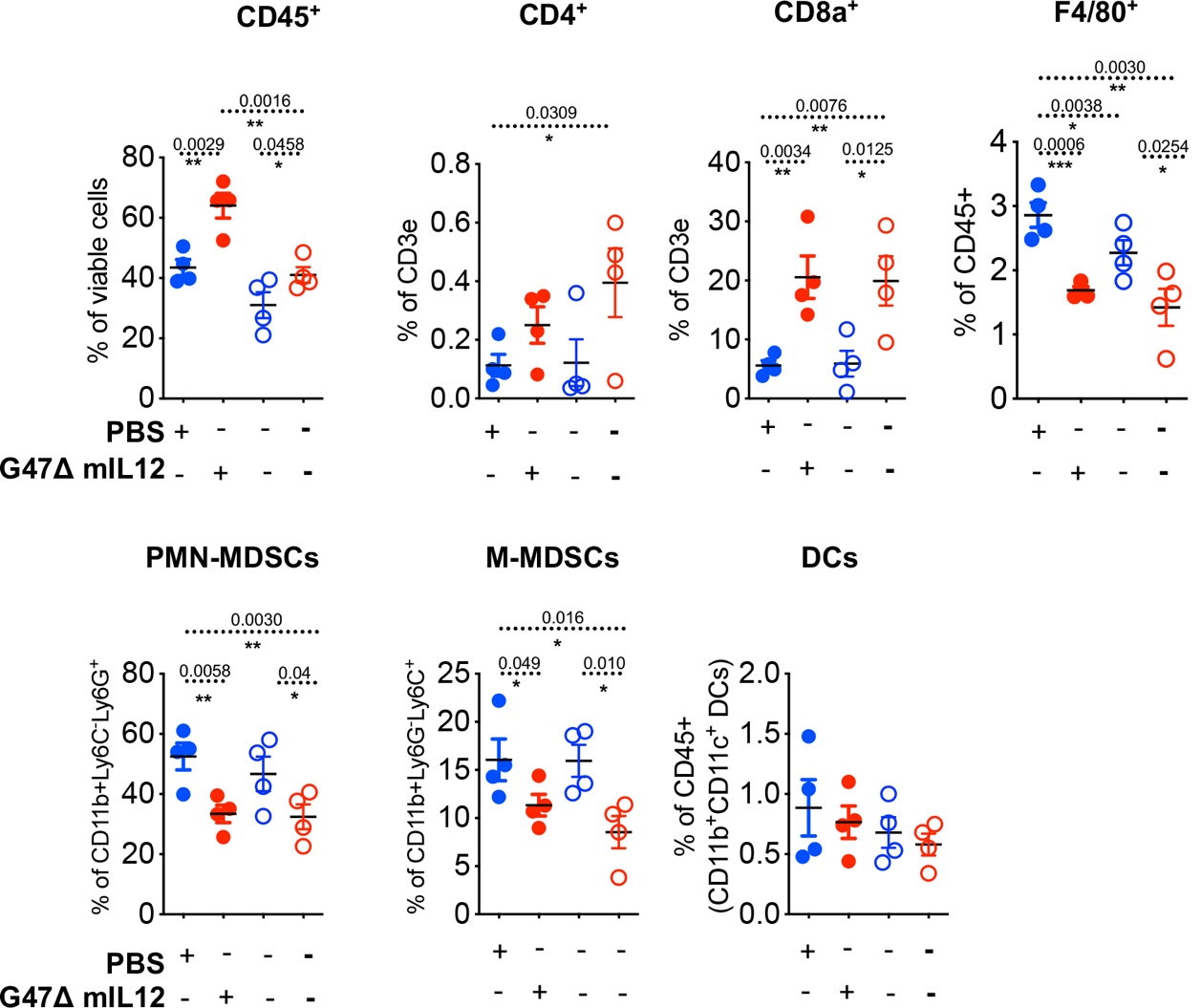


**Supplementary Figure 2. Immune cell population in treated and untreated tumors**. Same experiment as Figure 3. Filled dots represent treated tumors and empty dots represent untreated tumors. Mean ± SEM. Only statistically significant differences between groups are reported as *P*-values in the figures. Statistical significance was assessed by Student’s *t* test. * *P* = < 0.05, ** *P* = <0.01, *** *P* = < 0.001.

**Supplementary Figure 3**. **Validation of CD8a^+^ cell depletion in spleens of anti-CD8a antibody treated mice**. In vivo effects of anti-CD8a depletion antibodies on CD3e^+^CD4^+^ and CD3e^+^CD8a^+^ cell populations. Female BALB/c mice were injected twice at 3-day intervals with anti-CD8a antibodies (clone 2.43, 10 mg/kg, BioXCell) (n = 3) or isotype control rat IgG2b antibodies (n = 3) specific to keyhole limpet hemocyanin (clone LTF-2, 10 mg/kg, BioXCell) IP. Twenty-four hours after the last antibody injection, mice were sacrificed, spleen collected, splenocytes isolated and stained with or without anti-mouse CD4 and CD8a antibodies, and analyzed by flow cytometry. **(A)** Representative dot plots of spleen for Isotype control IgG- and anti-CD8a-treated mice (gated on live CD45^+^CD3e^+^ cells. Frequency of gated population is shown). **(B)** Mean percentage of CD8a^+^ T cells in IgG and anti-CD8a treated mice.
